# The Influence of Microenvironment on Survival of Intraportal Transplanted Islets

**DOI:** 10.3389/fimmu.2022.849580

**Published:** 2022-03-28

**Authors:** Ling-ling Yan, Li-ping Ye, Ya-hong Chen, Sai-qin He, Chen-yang Zhang, Xin-li Mao, Shao-wei Li

**Affiliations:** ^1^ Key Laboratory of Minimally Invasive Techniques & Rapid Rehabilitation of Digestive System Tumor of Zhejiang Province, Taizhou Hospital Affiliated to Wenzhou Medical University, Linhai, China; ^2^ Department of Gastroenterology, Taizhou Hospital of Zhejiang Province Affiliated to Wenzhou Medical University, Linhai, China; ^3^ Institute of Digestive Disease, Taizhou Hospital of Zhejiang Province Affiliated to Wenzhou Medical University, Linhai, China; ^4^ Health Management Center, Taizhou Hospital of Zhejiang Province Affiliated to Wenzhou Medical University, Linhai, China; ^5^ Taizhou Hospital of Zhejiang Province Affiliated to Wenzhou Medical University, Linhai, China

**Keywords:** islet transplantation, microenvironment, instant blood, mediated inflammatory reaction, inflammatory cytokine, therapeutic target

## Abstract

Clinical islet transplantation has the potential to cure type 1 diabetes. Despite recent therapeutic success, it is still uncommon because transplanted islets are damaged by multiple challenges, including instant blood mediated inflammatory reaction (IBMIR), inflammatory cytokines, hypoxia/reperfusion injury, and immune rejection. The transplantation microenvironment plays a vital role especially in intraportal islet transplantation. The identification and targeting of pathways that function as “master regulators” during deleterious inflammatory events after transplantation, and the induction of immune tolerance, are necessary to improve the survival of transplanted islets. In this article, we attempt to provide an overview of the influence of microenvironment on the survival of transplanted islets, as well as possible therapeutic targets.

## Introduction

Although type 1 diabetes cannot be prevented or reversed, islet transplantation—which restores insulin independence and prevents severe hypoglycemia—is currently proving to be a promising treatment (1, 2). However, the results of multiple clinical trials have shown that most transplant recipients fail to achieve complete insulin independence. Although the immunosuppressive regimen reported from Edmonton, Canada, has achieved unprecedented success in achieving insulin independence in islet transplantation (3), there are still some problems that affect the outcome of islet transplantation. Advancements in the acquisition of pancreatic islets, immunosuppression of islet recipients, and an increase in the number of transplanted islets are required (4). The average human pancreas has 300,000 to 1.5 million pancreatic islets, and only 60% of this islet cell mass is needed to maintain a normal glucose metabolism (5). However, 72% of islet recipients still require more than two successfully processed islet preparations to obtain a sufficient beta cell mass to compensate for islet death in the post-transplantation period (6). However, the source of pancreatic islets is limited.

It was observed that only 10% of recipients remain insulin dependent for more than 5 years and that most recipients re-use insulin because of a decline in the islet function over time (7). During transplantation, islet grafts are damaged by multiple challenges, including enzyme and mechanical damage caused by the isolation process, hypoxia, inflammation, immune rejection and toxicity of immunosuppressive drugs (8). The liver is currently the preferred transplantation site because the procedure is minimally invasive, easy to perform, and has low rates of bleeding and thrombosis (9). Early inflammatory responses strongly influence islet engraftment and survival after intrahepatic transplantation. This early immune response is triggered by immediate blood-mediated inflammatory response (IBMIR) and ischemia-reperfusion injury (10). Elevated inflammatory cytokines interleukin-1β (IL-1β), tumor necrosis factor α (TNF-α), interferon-γ (IFN-γ), were observed during islet transplantation, and macrophages also involved in regulating the cell injury of transplanted pancreatic islets (11). It was observed that concomitant transplantation of islets and vascular endothelial cells in diabetic rats can prolong the survival of islet grafts (12). Previous studies have found that cytokine inhibitors or drugs that inhibit the activation of liver macrophage activation can improve the function of pancreatic islets after transplantation (13, 14). Changes in the microenvironment, mainly involving inflammatory cytokines, endothelial cells, and immune cells, play a key role in the survival of islet grafts.

This review discusses the changes in the microenvironment (inflammatory cytokines, immune cells, endothelial cells, etc.) during clinical intraportal islet transplantation, and their influence on the survival of the islet grafts, with the aim of improving the success of clinical islet transplantation

## Status and Recent Progress of Clinical Intraportal Islet Transplantation

In 1990, David Scharp et al. reported the first case of type 1 diabetes allogeneic intraportal islet transplantation resulting in a short-term insulin independence, which opened the prelude to clinical islet transplantation (15). The International Islet Registry collected data from 267 islet allogeneic transplants from multiple centers between 1990 and 2001, only 12.4% of cases achieved insulin independence periods of at least 1 week, and 8.2% maintained insulin independence for more than 1 year. Appropriate immunosuppression remained a critical piece of the unsolved puzzle in order to improve long-term graft function and sustained insulin independence (16). Until 2000, Edmonton’s group reported that insulin independence was achieved in seven patients with type 1 diabetes who underwent islet transplantation. This protocol established the need for sufficient islet β cell mass for transplantation and also proposed a glucocorticoid-free immunosuppressive regimen (3). Since the publication of the Edmonton protocol, many countries around the world have successively carried out allogeneic islet transplantation for the treatment of type 1 diabetes. Extended follow-up of these trials sho wed a gradual loss of insulin independence over time with renewed need for exogenous insulin, with only 10% of patients showing insulin independence at 5 years after transplantation (7). Hering et al. reported in 2016 that the North American Clinical Islet Transplant Consortium conducted a multicenter, single-arm, phase 3 trial to further evaluate the efficiency of allogeneic islet transplantation. The assay, using product purified human pancreatic islets, achieved an HbA1c of 5.6% in the absence of severe hypoglycemia (17). A subsequent multicenter, open-label, randomized controlled trial in Europe reconfirmed the benefit of islet transplantation over intensive insulin therapy in patients with type 1 diabetes with severe hypoglycemia or after kidney transplantation (18). Recently, Marco et al. proposed that vitamin D alone or in combination with other anti-inflammatory agents may serve as a new immunomodulatory strategy to reduce the recurrence of autoimmune/allogeneic transplant rejection in intraportal islet transplantation, but its safety and efficacy further validation in large prospective studies is required (19). In intraportal islet transplantation model, islet pretreatment with mitomycin C prolonged graft survival by suppressing pro-inflammatory events and inducing latent regulatory lymphocytes (20). Xenogeneic and stem cell-derived islet tissues have entered early clinical trials, although much remains to be learned about the *in vivo* physiology and immunogenicity of various products. These advances provide more options for cellular therapy in diabetes treatment, providing an unlimited source of pancreatic islet tissue for future applications (21).

## Intraportal Islet Transplantation Challenges and Strategies

Instant blood mediated inflammatory reaction (IBMIR)

The IBMIR, which is triggered by exposed tissue factor on the islet surface and characterized by platelet activation and aggregation, and activation of the coagulation and complement systems, is a major obstacle after islet transplantation. Moreover, it increases the infiltration of neutrophils, monocytes and macrophages (13). These reactions are thought to cause the immediate loss of most of the transplanted islets (22), and may also increase the risk of islet rejection later through adaptive immune responses (23). Potential means for reducing islet inflammation and protecting islets can be achieved by the addition of anti-inflammatory agents, anticoagulants or coating islets with various protective macromolecules during islet culture (24). *In vitro* and *in vivo* experiments demonstrated that low molecular weight dextran sulfate prevented IBMIR, leukocyte infiltration was eliminated at high doses, and transplanted pig islets survived significantly longer in recipients treated with low molecular weight dextran sulfate (25). Another phase II, multicenter, active-controlled, randomized study, the Clinical Islet Transplant Consortium 01 study, revealed that systemic low molecular weight dextran sulfate treatment showed similar efficacy to heparin treatment in preventing IBMIR and promoting islet engraftment (26). However, the reason for the current reluctance to use it is that its target- activated partial thromboplastin time is 3 times longer than heparin’s (27). The use of biotin/avidin technology to bind preformed heparin complexes to the islet surface resulted in protection against IBMIR *in vitro* loop model and in an allogeneic porcine model of clinical islet transplantation (28). *In vitro* and *in vivo* data suggested that activated protein C exerted anti-inflammatory and anti-apoptotic activities by directly acting on cells after exposure of hepatic endothelial cells to pancreatic islets (29). However, the above remain to be verified by clinical studies (30, 31). And it has been confirmed that Cibinetide can reduce IBMIR related platelet consumption in a pro-inflammatory environment and protect isolated human islets (32). The evidence showed that α-1 antitrypsin inhibited IBMIR, which resulted in improved outcome of intraportal islet transplantation in mouse model (33). Currently, the addition of heparin is the standard approach but it is insufficient (27).

## Complement system

Complement activation is triggered by natural immunoglobulin (Ig)G or IgM. When isolated islets are exposed to blood, the complement system is rapidly activated and causes lysis of islet cells (34). Complement activation occurs *via* the classical pathway and an alternative pathway, which leads to the formation of a complex composed of C5b-9, which forms a “channel” through the cell membrane, leading to cell lysis and death. Another major function of the complement system is the production of anaphylatoxins C3a and C5a, which enhance the inflammatory response to islets (23). C3a and C5a produced by complement activation are powerful chemoattractants for macrophages and neutrophils (10). Activation through the C5a receptor can cause granulocytes to release enzymes, such as myeloperoxidase and elastase, and promote monocytes to release cytokines, such as IL-1, IL-6, IL-8, and TNF-a. C5a stimulates endothelial cells to release heparin sulfate, upregulate tissue factor, secrete von Willebrand factor and express P-selectin, which is conducive to fibrin deposition and which enhances thrombin-mediated platelet aggregation and polymorphonuclear leukocyte adhesion (23). Tissue factor and many adhesion molecules can be expressed by endothelial cells triggered by soluble C5b-9 (sC5b-9) (35). Complement activation may therefore induce direct inflammation and indirect effects mediated by endothelial cells.

In allogeneic transplantation, C3 is one of the important factors triggering rejection in mice (36, 37) and humans (38). Numerous studies have shown that the combination of C3 fragment C3dg with an antigen can be used as a strong adjuvant to promote both cellular and humoral responses (39). Therefore, it can be reasonably expected that complement activation and C3dg binding will trigger an immune response, leading to an adaptive immune response to the graft (34). Therefore, small interfering RNA (siRNA) targeting C3 and C5a receptors may increase the viability of transplanted islets (40).

## Hypoxic damage

Islets are easily damaged under hypoxic conditions prior to transplantation (including pancreas procurement, islet isolation and culture) and exposure to hypoxic environment of the transplant site after transplantation (41). Hypoxia damages islet β cell function, which manifested anaerobic glycolysis, showed elevated lactate and reduced responsiveness to high glucose levels (42). The decrease of blood oxygen partial pressure can lead to irreversible β cell dysfunction, resulting in higher fasting blood-glucose and lower C-peptide levels (43). Calcium influx into islet cells has also been shown to cause cell damage in rat and human islets cultured under hypoxic conditions (44). In order to cope with the impact of hypoxia on the quality and function of islets, the following explorations were attempted: (1) Compared with static cold storage, perfusion effectively reduced anoxic death of islet cells, and islet production was higher after perfusion (45, 46). Perfluorohexyloctan, a semi-fluorinated liquid fluorocarbon, maintained higher intrapancreatic pO2 and improved islet viability and function with porcine pancreas (47, 48). In addition, oxygen supply can be increased and oxygenation of islets can be improved by decreasing the culture density of islets before transplantation (46, 49). (2) Photosynthesis of thermostable microalga (Chlorella sorokiniana) was applied as a method to supply oxygen to cultured islets coencapsulating in alginate gel (50). Microparticle-mediated-oxygenation has been studied to improve islet transplantation.Co-transplantation of oxygen-generating microparticles and minimal islet mass within fibrin-conjugated heparin/VEGF collagen scaffold has enormous potential to enhance islet revascularization, diabetes reversal and oxygenation (51). (3) As mentioned above, the influx of calcium ions into islet cells can also induce islet injury. It was found that potassium channel activator (diazoxide) and calcium channel blocker (nifedipine) were helpful to restore the synthesis of insulin protoplasts and islet cell necrosis caused by hypoxia when used as preconditioning agents (44, 52). (4) Curcumin has the ability to protect β cells from hypoxia damage (53). Puerarin could alleviate β cell apoptosis and malfunction by hypoxic injury of β cells in corpulent mice induced by cobalt chloride induced *via* PI3K/Akt pathway activation (54). (5) Reconstructing the capillary network in islets is very important to prevent hypoxia and preserve function. Previous studies have shown the effectiveness of prevascularization of the graft bed for subcutaneous islet graft survival (55–58). The use of islet-cell cluster in clinical islet transplantation may be a strategy to prevent islet loss caused by hypoxia after transplantation (59).

## Immunosuppressive drug toxicity and immune tolerance induction

During intraportal islet transplantation, isolated islets are exposed to high levels of immunosuppressive drugs, which are detrimental to islet engraftment/survival and long-term function (60). Previously, the standard protocol of immunosuppression for islet transplantation included a combination of corticosteroids, calcineurin inhibitors (tacrolimus and cyclosporine), and purine analogs (mycophenolate mofetil). Many of the above drugs had been shown to be diabetogenic, impairing insulin secretion (61). The Edmonton protocol in 1999 took a major step forward in islet transplantation with the introduction of steroid-free therapy based on low-dose sirolimus, tacrolimus, and daclizumab (3). Although the results of multicenter clinical trials suggested that this protocol could provide short-term insulin independence and reduce the incidence of acute rejection (62), patients receiving long-term immunosuppressive drugs are susceptible to multiple adverse effects, such as infections (63), malignancies (64), *de novo* diabetes (65) and organ toxicities (66).

Immune tolerance induction is a promising strategy to accept histocompatibility complex (MHC)-mismatched allografts without reducing resistance to infection or increasing other complications (67). For immunological tolerance to allografts, the high proportion of MHC alloreactive T cell is considered as a major barrier to tolerance induction. Central T cell tolerance refers to the deletion of reactive clones in the thymus during negative selection. Peripheral T cell tolerance includes peripheral deletion, anergy/exhaustion, and suppressive function of regulatory T cells (Treg) (68). At present, how to apply the inherent immune tolerance mechanism of the human system to induce donor-specific immune tolerance is the key to solving transplant rejection (69). Singh et al. reported that apoptotic donor lymphocyte infusion prior to transplantation induced long-term tolerance (>1 year) of islet grafts in a non-human primate (NHP) model, which had made a breakthrough in the tolerance induction protocol for allogeneic islet transplantation (70).

Intrathymic inoculation of recipient APCs pulsed with allopeptides can induce intrathymic tolerance, however it is an invasive technique and the thymus regresses with age and has limited potential in adults (71). Another potentially more effective approach to achieving central tolerance is the generation of hematopoietic chimerism or mixed allogeneic chimerism, with lethal total body irradiation or sublethal total body irradiation (assisted by anti-CD4, CD8 monoclonal antibodies or costimulatory blockade) in the prospective transplant recipients, in order to make room for the transplanted bone marrow. Bone marrow cell transplantation can reconstitute the recipient’s hematopoietic compartment with donor hematopoietic stem cells, inducing donor-specific tolerance to islet allografts (62, 72). Another strategy to induce tolerance is to deplete alloreactive T cells prior to transplantation, promoting a hyporesponsive environment that drives tolerance transition (73). T cell depletion can be achieved by total body irradiation, lymphocyte depleting alloantibodies. Among them, antithymocyte globulin (ATG) is a potent inducer of T cell depletion, and ATG alone or in combination with other drugs can prolong the survival of allografts (74, 75). Other pathways for inducing immune tolerance include costimulatory signal blockades, induction and expansion of regulatory T (Treg) cells, etc (62). And in the intrahepatic mouse allogeneic islet transplantation model, Lee et al. demonstrated for the first time that short-term single administration of anti-CD154 monoclonal antibody could induce FoxP3+ Treg cell-mediated immune tolerance (67).

## Key Inflammatory Factors and Cells Associated With Islet Cell Dysfunction

Several mediators have been found to cause islet dysfunction and/or cellular death after islet transplantation, including inflammatory cytokines (IL-1β, TNF-α, and IFN-γ), nitric oxide (NO), and nitric oxide synthase (iNOS) (76).

## IL-1β

**Figure 1:**
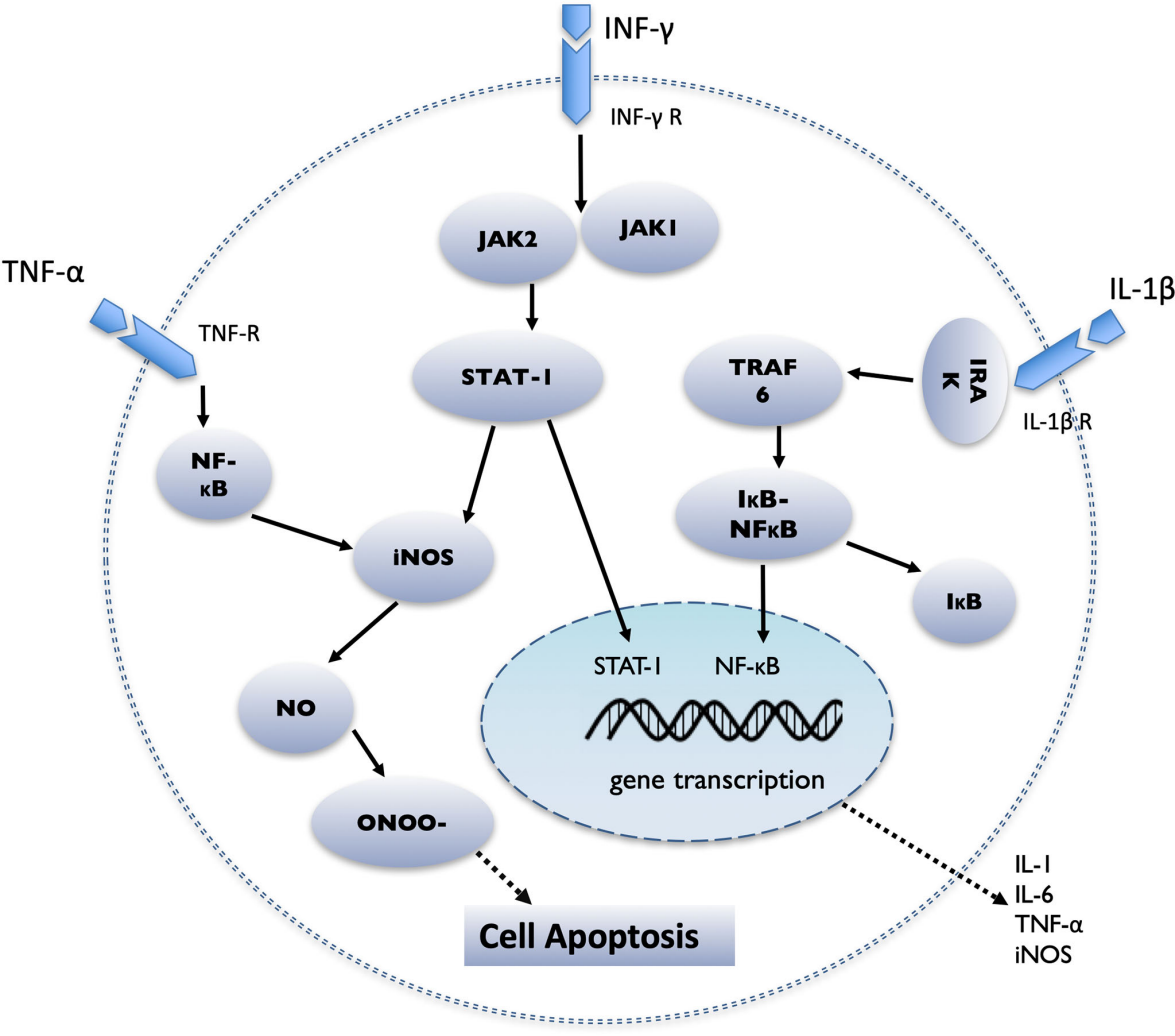
Schematic diagram demonstrating the intracellular cascade of events that occur in the pancreatic islet after stimulation by IL-1β, IFN-γ and TNF-α. IL-1β binds to the IL-1β receptor (IL-1βR), causing TNF receptor-related factor 6 (TRAF6) to be activated by IL-1 receptor-related kinase (IRAK), then NF-κB is released from inhibitory IκB, transferred from the cytoplasm to the nucleus, and regulates the transcription of various genes, including IL-1, IL-6, TNF-α, and iNOS; TNF-α binding to the TNF receptor (TNF-R), initiates the activation of NF-κB and the induction of apoptosis; IFN-γ binds to the IFN-γ receptor (IFN-γR) and causes the activation of JAK1 and JAK2. JAK2 then activates signal transducers and activators of transcription 1 (STAT1), which then transfers to the nucleus, where it performs gene regulation.

IL-1β is one of the most important mediators of islet injury and plays an important role in the process of pancreatic islets dysfunction, which may represent an early inflammatory marker of graft failure (77). IL-1β is secreted by Kupffer cells, islet resident macrophages, and neutrophils around the transplantation site (78). IL-1β secretion increases during islet acquisition, islet isolation, islet culture, and islet transplantation (77). IL-1β binds to the IL-1β receptor (IL-1βR) on the surface of pancreatic islet cells, causing TNF receptor-related factor 6 (TRAF6) to be activated by IL-1 receptor-related kinase (IRAK), which in turn leads to the phosphorylation and degradation of IκB. Then NF-κB is released from inhibitory IκB, transferred from the cytoplasm to the nucleus, and regulates the transcription of various genes, including IL-1, IL-6, TNF-α, and iNOS (76, 79) (**Figure 1**). Activation of iNOS results in the production of NO, which is directly related to β-cell apoptosis (80).

The damage of islet cells starts from the donor. Although most pancreatic islet transplants use organs from heart-beating brain-dead (BD) donors, acute physiological changes after brain death of BD donors may still cause significant damage to islets from inflammatory events. Brain death can stimulate various cells to produce pro-inflammatory cytokines, and produce a so-called “cytokine storm”, including IL-1β in BD donors, which greatly reduces the islet yield, functionality, vitality, and engraftment after transplantation (8). One study has shown that the administration of exendin-4 to BD donors can reduce the expression of IL-1β, thereby increasing both the islet viability and insulin secretion in the pancreas after glucose stimulation in a BD rat model (81). In the BD rat model, treatment with a selective neutrophil elastase inhibitor, sivelestat sodium, decreased the expression of IL-1β, significantly improved the islet yield and function *in vitro*, and suppressed hypercytokinemia-mediated beta-cell death (82).

Then, in the process pancreas digestion and islet purification, enzymatic and mechanical stress can induce inflammatory mediators, such as IL-1β, in the islets (77). The islet basement membrane is lysed during the digestion and separation of the pancreas, which interrupts the communication between islet basement membrane proteins and the integrins expressed by the islets (83). The loss of the interaction between the internal cells and the external microenvironment also interrupts the transmission of pro-survival signals (84). The isolation of pancreatic islets eventually leads to the increased expression of many stress kinases, which subsequently activates pro-inflammatory and pro-apoptotic pathways (43, 85). Furthermore, the functional clustering of differentially expressed genes revealed the upregulation of genes related to cell growth, angiogenesis, inflammation, and apoptosis after isolation and culture (43). A study has shown that islets in early culture (2 days) express more genes, including IL-1β, than islets in long-term culture (7-11 days). It seems that culturing islets before transplantation is beneficial for reducing the expression of inflammatory mediators (86). However, in cultured adult porcine islets, IL-1β mRNA was continuously detected at 1, 4, 8, and 11 days after isolation, and slightly increased over time (87). It is worth noting that a pretreatment culture with anakinra (IL-1 receptor antagonist) prior to human islet transplantation can improve the survival and function of human islets during culture (88).

The early damage of pancreatic islets is mainly manifested by an IBMIR, leading to a cytokine storm, involving IL-1β, TNF-α, and IFN-γ. These cytokine-activated macrophages produce IL-1β, which triggers the cytokine storm at the transplantation site, leading to a negative chain of events (89). Syngeneic transplant models have shown that the nonspecific inflammatory response increases IL-1β at the transplant site and affects early graft failure. In that study, the expression of IL-1β mRNA was maximal on day 1 after transplantation and then declined towards pre-transplantation levels on day 7 (90). Previous animal studies have indicated that approximately 60% of islet grafts lose their function *via* non-specific inflammation within 3 days after transplantation (91, 92). The overexpression of IL-1β receptor antagonist protein in transplanted islets can improve the outcome of the transplantation (93).

## TNF-α

In humans, it appears that IL-1β must act in combination with IFN-γ and/or TNF-α (76). After TNF-α binds to the TNF receptor (TNF-R), it forms a trimer and undergoes conformational changes, which leads to the exposure of the intracellular death domain, and initiates the activation of NF-κB, the activation of the MAPK pathway, and the induction of apoptosis (40). Wen et al. revealed that TNF-α was significantly elevated in patients following allogeneic islet cell infusion compared with patients receiving autologous transplantation (94). Recently, multiple studies have shown that after pancreatic islet allotransplantation, the early use of a combination of anti-IL-1β (anakinra) and TNF-α (etanercept) inflammation blockade is beneficial for reducing islet damage caused by nonspecific inflammation and presumably led to better engraftment (95) (**Figure 1**).

## IFN-γ

IFN-γ binds to the IFN-γ receptor (IFN-γR) and causes the activation of JAK1 and JAK2. JAK2 then activates signal transducers and activators of transcription 1 (STAT1), which then transfers to the nucleus, where it performs gene regulation (96). One study showed that IFN-γ transcripts were found in allografts at 1, 3, 5, and 7 days after transplantation, and peaked on day 5, but there were no such cytokines in syngeneic grafts (97). Some studies have shown that combined cytokines such as IL-1β, TNF-α, and IFN-γ lead to islet cell dysfunction or death (98).

## NO and Inducible Nitric Oxide Synthase

Cytokines, such as IL-1β, TNF-α and IFN-γ, mainly stimulate the large expression of iNOS in β cells and macrophages to synthesize excessive NO, thereby causing damage to the pancreatic islets (99); the latter can form a highly active free radical peroxynitrite (ONOO-) by losing an electron and combining with superoxide free radicals, which have strong cytotoxicity and promote apoptosis (100, 101) (**Figure 1**). NO affects many physiological β-cell processes, including inhibition of oxidative metabolism, changes in the expression of target genes, inhibition of glucose-stimulated insulin secretion, damage to DNA, and induction of endoplasmic reticulum (ER) stress. If exposure to NO is prolonged, it activates various signal cascades and eventually leads to the death of β cells (102). In xenogeneic islet transplantation animal models, selective iNOS inhibitors can suppress the production of induced NO, to prevent early islet graft failure (103).

## Immune Cells

### Macrophages

Macrophages, which are one of the cellular components of the innate immune system, show great heterogeneity in physiological and pathological conditions. They can be polarized into pro-inflammatory macrophages (M1) or anti-inflammatory macrophages (M2) in different environments (104). Lipopolysaccharide and interferon IFN-γ can activate M1 macrophages to secrete TNF-α, iNOS and superoxide anion to play pro-inflammatory and host defense functions. In contrast, M2 macrophages have a protective role in the immune response and inflammation. Other studies showed that M2 macrophages can be activated by interleukin (IL)-4 and IL-13, and play an immune regulatory and anti-inflammatory role, through the secretion of IL-1Rα, IL-1,TNF-α, IL-10, and other cytokines (104, 105).

**Figure 2:**
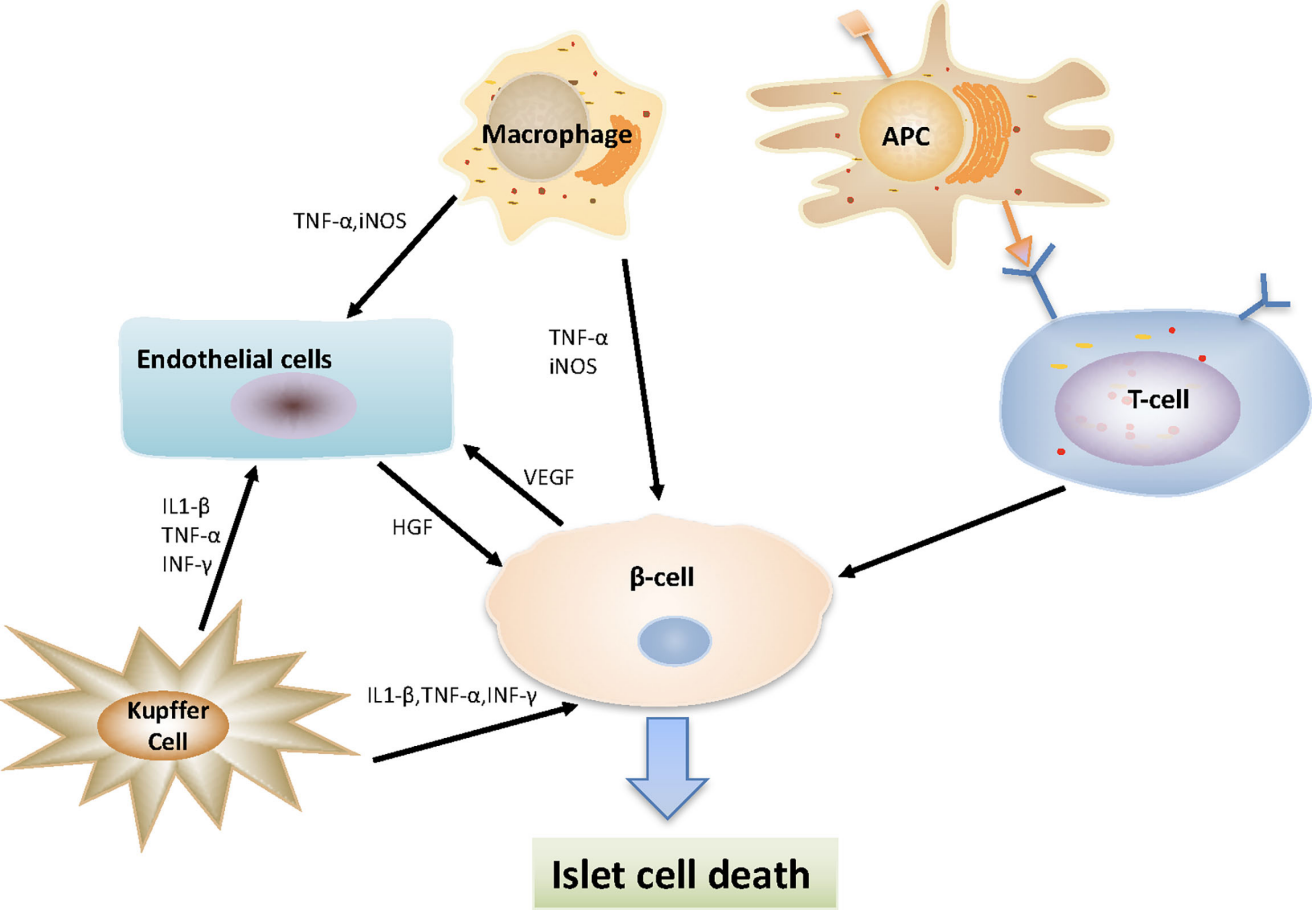
Interaction between various types of cells after islet transplantation. Macrophages secreting TNF-α and iNOS, which act on beta-cells and endothelial cells to play a pro-inflammatory function; Kupffer cells secreting a series of substances (including IL-1β, IFN-γ and TNF-α), directly affecting the survival of intrahepatic islets and endothelial cells; Antigen presenting cells (APCs) take and process antigens from donor and present antigens to host T cells, then reactive CD8+, CD4+ T-cells destroy transplanted islet β cells.

After the islets are infused into the portal vein, the activated liver-resident macrophages (Kupffer cells) play a central role in the inflammatory responses within the liver, secreting a series of factors (including arachidonic acid metabolites, TNF-α, IFN-γ, IL-1, IL-6, complement, coagulation factors, reactive oxygen species and nitrogen) to recognize and respond to various signals from the surrounding microenvironment, directly affecting the survival of intrahepatic islets (11, 106, 107). Furthermore, Kupffer cells can induce a response of other non-immune cell subsets, including endothelial cells (108). In addition, it has been suggested that ischemia reperfusion phenomena may directly trigger the activation of sinusoidal endothelial cells and help trigger nonspecific inflammatory responses (109). The transplanted islets, residual endotoxin produced in the process of islet isolation, and liver sinusoidal endothelial damage can all activate Kupffer cells (11, 110). In addition, tissue damage can recruit inflammatory macrophages (M1), which in turn cause islet damage (111). At the same time, dying β cells also produce high mobility group box 1 (HGMB) and iNOS, attracting more macrophages to the liver, and enhancing inflammation and β cell death (14, 112) (**Figure 2**).

Macrophage depletion can significantly reduce the expression of IL-1β and TNF-α, indicating the role of macrophages in the production of inflammatory cytokines (11). Gou et al. demonstrated that Alpha-1 antitrypsin can protect the survival of islet grafts, in part by inhibiting the polarization of M1 macrophages both *in vivo* and *in vitro* (14). One study has mentioned that the local sustained-release of dexamethasone in grafts promotes the survival of mouse pancreatic islet grafts by inducing the differentiation of M2 macrophages in the graft microenvironment as well as the secretion of anti-inflammatory factors (104). Chappell et al. showed that activated M2 macrophages could improve the survival conditions of grafts in a mouse model by improving revascularization or neovascularization, which had a repair function in the graft reaction (113).

### Lymphocytes

Type I diabetes is characterized by the autoimmune-mediated damage of islet β-cells, and the transplanted islets will also be attacked by the same stresses that destroy host β-cells (40). CD8+ T cells and CD4+ T cells are the major players in the destruction of β cells. Activated T cells produce cytokines such as IFN-γ, TNF-α, and lymphotoxin to induce β cell apoptosis. T cells also express ligands for the Fas receptor and TNF-related apoptosis-inducing ligands, both of which lead to apoptosis by activating effector caspases. In addition, CD8+ T cells directly contact and promote the release of granzyme B into the cytoplasm of target cells through perforin, thereby activating nucleases and caspases to kill the target cells (40, 98). In some studies, CD8+ T cell infiltration was observed in the pancreas of type 1 diabetic patients and the transplanted pancreas (114, 115). In mouse models, deficiency of CD4+ T cells was observed to stop progression to insulitis (116).

During the rejection of allogeneic transplantation, the host immune response can directly or indirectly be activated by T cells recognizing the donor tissue. Direct graft recognition involves an interaction between the donor tissue resident antigen-presenting cells (APCs) and host T cells *via* major histocompatibility complex (MHC) (117). Indirect recognition involves the treatment of donor graft peptides by host APCs and corresponding MHC interactions to stimulate host T cells (118). APCs, including macrophages, dendritic cells (DC), passenger leukocytes, from both donors and host are involved in the antigen presentation (40). The activation and maturation of T cells depends on the signals from the above APCs. If these signals are blocked, T cells will undergo apoptosis (**Figure 2**).

DCs can not only initiate an immune response, but also induce central or peripheral immune tolerance (40). Mature or activated DCs can initiate a positive immune response, while immature DCs or DC precursors show tolerance. 
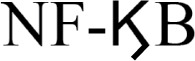
 is a transcription factor that is necessary for DC differentiation and maturation. The inhibition of the 
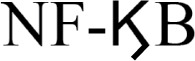
 pathway has been shown to produce tolerogenic DCs (119). RelB is a major 
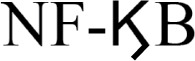
 protein, which inhibits the expression of MHC-II, CD80, and CD86, and ultimately prevents the maturation of dendritic cells. RelB silenced DCs can inhibit antigen-specific alloreactive immune rejection and reduce the proliferation of antigen-specific T cells (120). In another study, tolerogenic DCs were generated by inhibiting the expression of CD80 or CD86. The administration of modified dendritic cells (DC) can prolong the survival of allografts, thereby inducing T cell hyporesponsiveness and apoptosis (121).

### Granulocytes

The poor outcome of intraportal islet transplantation can be explained by IBMIR, one of which is characterized by leucocyte infiltration. The islets were mixed with ABO-compatible blood in a heparinized tube, and the first neutrophilic granulocytes appeared in the islets after 15 minutes, increasing at 1 hour and peaking at 2 hours (122). Neutrophilic granulocytes induce cell damage through cytotoxic attack and phagocytosis. After neutrophil activation, superoxidase is produced to form reactive oxygen species (ROS) and release protease, both of which are involved in killing microbes (123). Neutrophilic granulocytes are also known to contain a large number of cytokines, which are released upon activation, and there is much evidence that cytokines have a damaging effect on pancreatic islets (124). And their infiltration results in the release of chemokines such as TNF-α and macrophage inflammatory protein 1α (MIP-1α) from T cells and macrophages. The mobilization of this immune effector may have effects on specific immune systems, inducing and enhancing cellular rejection (122). The massive infiltration of neutrophilic granulocytes not only causes functional impairment or reduces the mass of the implanted islets, but may also amplify the subsequent immune response, causing direct damage to the islets (125). Therefore, the development of drugs targeting neutrophil toxicity may markedly improve the outcome of intraportal islet transplantation.

### Endothelial Cells

Pancreatic islets have an extensive capillary network, which—in addition to providing nutrients and oxygen to the islet endocrine cells and transporting hormones to the peripheral circulation—is an important source of signals for improving the survival rate and function of islet β cells (126). While islets only constitute 1% of the weight of the pancreas, they receive approximately 15% of the blood flow of the pancreas (127). In the process of the isolation, the islets are disrupted from the surrounding tissue and the capillary networks. Therefore, revascularization of the islets after transplantation is important for the function and survival of the islet graft. The endothelial cells retained in the islets after islet isolation are lost after the islets are cultured for 7 days. The isolated islets are considered to be an avascular tissue, and revascularization is carried out through the blood vessels that grow from the host organ to the islets (128). Angiogenesis begins on the first day after transplantation, and vascular remodeling may continue for up to 3 months (129). In comparison to cultured islets, transplantation of freshly isolated islets containing numerous endothelial cells may significantly improve the vascularization of transplanted islets, which in turn leads to an enhanced endocrine function and the survival of islet grafts (130). Olsson et al. observed that islet grafts obtained from freshly isolated islets have higher vascular density and oxygen tension, as well as higher ability to cure chemically induced diabetes, in comparison to islet grafts obtained from cultured islets (131).

Pancreatic islets and vascular endothelial cells secrete high levels of vascular endothelial growth factor (VEGF), which can recruit neovascularization (132). Cheng et al. used adenovirus containing cDNA from human VEGF isoforms to transfected islets and transplanted it into diabetic nude mice. It was found that the blood glucose was normal and that the revascularization of islets was improved (133). Johansson et al. demonstrated that the production of hepatocyte growth factor (HGF) *in vitro* by endothelial cells increased the proliferation of beta cells, which in turn required VEGF from beta cells (134). VEGF can also stimulate the release of interleukins and increase blood flow to ischemic tissues (135) (**Figure 2**). However, the supplementation of VEGF in islet grafts may have a negative impact, which recruits and amplifies inflammation, which may destroy islets (133). Previous *in vitro* and *in vivo* studies have shown that resident liver macrophages and endothelial cells can mediate early islet dysfunction by secreting cytokines and activating inducible iNOS (103).

## Conclusion

Islet transplantation remains a promising treatment to improve the quality of life for many individuals with type 1 diabetes. If the restoration of normal glucose tolerance can be achieved, non-specific inflammation during transplantation can be reduced, and immune tolerance to islet tissue can be induced, it will be an ideal treatment for this disease. However, during the process of islet transplantation—including isolation, culture, and islet implantation—inflammation, ischemia, hypoxia, and immune responses will occur, resulting in the loss of the graft. The transplantation microenvironment plays an important role. Cytokine-mediated non-specific inflammation, immune cell-mediated rejection, and endothelial cells participate in post-transplant vascular remodeling, which directly and indirectly affect graft survival. Some cytokine inhibitors and siRNA targeting complement receptors have been shown to improve the viability of transplanted islets. Understanding the influence of the microenvironment on the survival of transplanted islets, as well as possible therapeutic targets is significant for the future of islet transplantation. The improvement of the microenvironment and the continuous progress of transplantation strategies will eventually improve the prognosis of transplant recipients.

## Author Contributions

All authors contributed to the writing and editing of the manuscript and contributed to the article and approved the submitted version.

## Funding

This work was supported in part by Medical Science and Technology Project of Zhejiang Province (2021PY083), Program of Taizhou Science and Technology Grant (20ywb29), Major Research Program of Taizhou Enze Medical Center Grant (19EZZDA2), Open Project Program of Key Laboratory of Minimally Invasive Techniques & Rapid Rehabilitation of Digestive System Tumor of Zhejiang Province (21SZDSYS01, 21SZDSYS09) and Key Technology Research and Development Program of Zhejiang Province (2019C03040).

## Conflict of Interest

The authors declare that the research was conducted in the absence of any commercial or financial relationships that could be construed as a potential conflict of interest.

## Publisher’s Note

All claims expressed in this article are solely those of the authors and do not necessarily represent those of their affiliated organizations, or those of the publisher, the editors and the reviewers. Any product that may be evaluated in this article, or claim that may be made by its manufacturer, is not guaranteed or endorsed by the publisher.
